# Colonic Absorption of Low-Molecular-Weight Metabolites Influenced by the Intestinal Microbiome: A Pilot Study

**DOI:** 10.1371/journal.pone.0169207

**Published:** 2017-01-25

**Authors:** Mitsuharu Matsumoto, Takushi Ooga, Ryoko Kibe, Yuji Aiba, Yasuhiro Koga, Yoshimi Benno

**Affiliations:** 1 Dairy Science and Technology Institute, Kyodo Milk Industry Co. Ltd., Hinode-mach, Tokyo, Japan; 2 Benno Laboratory, Innovation Center, RIKEN, Wako, Japan; 3 Human Metabolome Technologies, Inc., Tsuruoka, Japan; 4 Department of Infectious Diseases, Tokai University School of Medicine, Isehara, Japan; University of Illinois at Chicago, UNITED STATES

## Abstract

Low-molecular-weight metabolites produced by the intestinal microbiome play a direct role in health and disease. However, little is known about the ability of the colon to absorb these metabolites. It is also unclear whether these metabolites are bioavailable. Here, metabolomics techniques (capillary electrophoresis with time-of-flight mass spectrometry, CE-TOFMS), germ-free (GF) mice, and colonized (Ex-GF) mice were used to identify the colonic luminal metabolites transported to colonic tissue and/or blood. We focused on the differences in each metabolite between GF and Ex-GF mice to determine the identities of metabolites that are transported to the colon and/or blood. CE-TOFMS identified 170, 246, 166, and 193 metabolites in the colonic feces, colonic tissue, portal plasma, and cardiac plasma, respectively. We classified the metabolites according to the following influencing factors: (i) the membrane transport system of the colonocytes, (ii) metabolism during transcellular transport, and (iii) hepatic metabolism based on the similarity in the ratio of each metabolite between GF and Ex-GF mice and found 62 and 22 metabolites that appeared to be absorbed from the colonic lumen to colonocytes and blood, respectively. For example, 11 basic amino acids were transported to the systemic circulation from the colonic lumen. Furthermore, many low-molecular-weight metabolites influenced by the intestinal microbiome are bioavailable. The present study is the first to report the transportation of metabolites from the colonic lumen to colonocytes and somatic blood *in vivo*, and the present findings are critical for clarifying host-intestinal bacterial interactions.

## Introduction

The intestinal microbiome plays an important role in health and disease [1] because it influences pathological and normal homeostatic functions involved in obesity [2, 3], immune disease [4, 5], colon cancer [6], brain function [7], behavior [8], and life-span [9, 10]. We propose that these interactions depend upon direct stimulation of bacterial cell components and the effects of bacterial metabolites. Bacterial cell components influence the physiological and pathological functions of the immune system through the direct stimulation of Toll-like receptors expressed by colonocytes and dendritic cells that reside in the colonic mucosa [11, 12]. In contrast, the relationships between bacterial metabolites and health/disease are not well known, except for specific metabolites such as short-chain fatty acids (SCFA) [13]. It is also unclear what metabolites are transported to the body from the colonic lumen. Although many blood metabolites have been reported to be different between GF and Ex-GF mice [14], no comprehensive data are available to indicate that blood and colonocytes contain bacterial metabolites derived from the colonic lumen because appropriate analytical methods are not available. There is a general concept that the role of the colon is mainly to absorb water, electrolytes, and some vitamins; thus, in the field of gastrointestinal physiology and nutrition, there have been few reports indicating that low-molecular-weight chemicals are absorbed from the large intestine, and physiologists and nutritionists have typically not considered metabolites produced by intestinal bacteria as research targets. In this study, we tried to identify the bioavailable low-molecular-weight metabolites transported to middle colonic tissue and/or blood from the colonic lumen.

**Fig 1:**
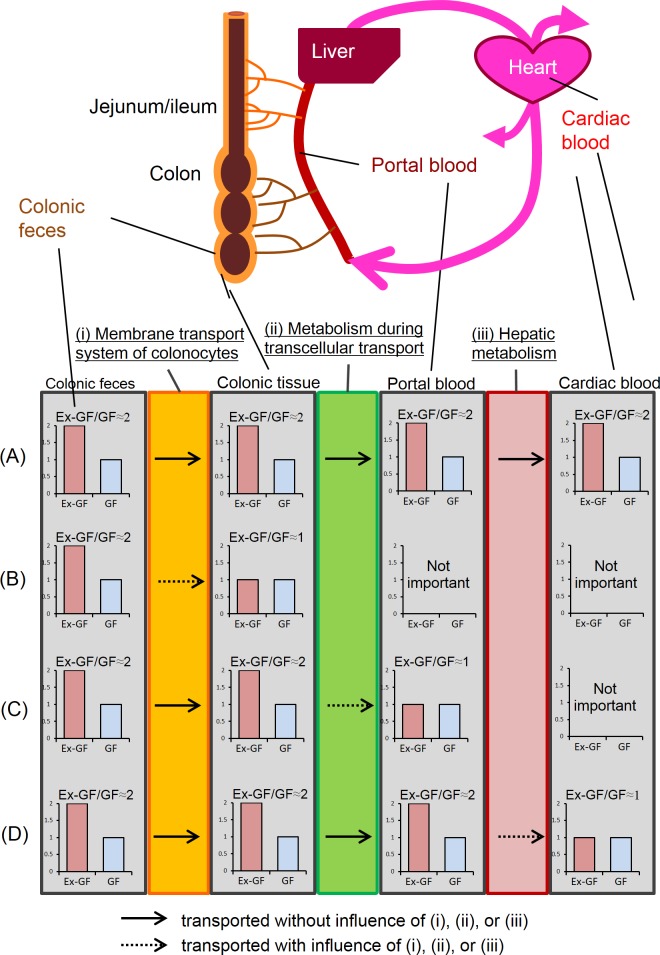
Estimation of metabolites to transport to body from colonic lumen using the difference of concentration between GF and Ex-GF mice (Ex-GF/GF ratio). (A) Metabolites with similar Ex-GF/GF ratios in colonic feces, colonic tissue, portal plasma, and cardiac plasma were considered to be transported from the colonic lumen to cardiac blood. (B) Metabolites with different Ex-GF/GF ratios between colonic feces and colonic tissue are controlled by (i) membrane transport system (C) Metabolites with different Ex-GF/GF ratios between colonic tissue and portal plasma are controlled by (ii) metabolism during transcellular transport. (D) Metabolites with different Ex-GF/GF ratios between portal plasma and portal.

It is theoretically possible for transportation of specific substances to be detected using stable or radioactive isotopes [15, 16]; however, it is impossible to comprehensively detect a large number of metabolites using these methods. Using capillary electrophoresis combined with time-of-flight mass spectrometry (CE-TOFMS), which analyzes and differentially displays metabolic profiles [17], we previously demonstrated that 125 low-molecular-weight metabolites that showed significant differences in the colonic feces of germ-free (GF) and ex-germ-free (Ex-GF) mice (i.e., mice that were previously GF-free but now harbored specific pathogen-free mouse intestinal microbiota) were influenced by the intestinal microbiome [18]. Here, we focused on these differences between GF and Ex-GF mice to determine the identities of low-molecular-weight metabolites transported to body from colonic lumen. In brief, the blood Ex-GF/GF ratio of metabolites that are transported to the blood from the colonic lumen is similar to the Ex-GF/GF ratio in colonic feces (Fig 1). We determined the Ex-GF/GF ratio for each metabolite derived from the colonic feces, colonic tissue (colonocytes), portal plasma, and cardiac plasma of GF and Ex-GF mice and searched for metabolites with similar Ex-GF/GF ratios in the colonic feces and colonic tissue, portal plasma, and cardiac plasma.

## Materials and Methods

### Mice and diets

Germ-free (GF) BALB/c mice were purchased from Japan Clea Inc. (Tokyo, Japan) and bred at the Department of Infectious Diseases, Tokai University School of Medicine, Kanagawa, Japan. Six male litters were divided into GF and Ex-GF (see below) groups to remove effects caused by inherited individual distinctions. Mice were housed in Trexler-type flexible film plastic isolators with sterilized tips (CLEA Japan, Inc., Tokyo, Japan) as bedding. The mice were provided with water and commercial CL-2 pellets (CLEA Japan, Inc.) that were sterilized using an autoclave (121°C, 30 min). Bacteriological contamination of feces was analyzed throughout the cultivation procedure using Gifu Anaerobe Medium (GAM) agar (Nissui, Tokyo, Japan). Using a gastric gavage tube, we inoculated the stomachs of Ex-GF mice (4 weeks of age) with 0.5 mL of a 1:10 dilution of feces obtained from specific-pathogen-free BALB/c mice and housed them mice of various for 3 weeks until collecting specimens at 7 weeks of age. This study was carried out in strict accordance with the recommendations in the guidelines of the Animal Care Committee of Tokai University. The protocol was approved by the Kyodo Milk Animal Use Committee (Permit Number: 2009–03).

### Specimen preparation

Cardiac and portal blood samples were collected from mice anesthetized with inhalation of isoflurane using small animal anesthetizer MK-A110 (Muromachi Kikai Co. Ltd., Tokyo, Japan) (7 weeks of age) into tubes containing sodium ethylenediamine tetraacetate (final concentration, 0.13%). Portal blood (50–100 μl) and cardiac blood (50–100 μl) were collected using a needle. The blood samples were centrifuged for 20 min at 2,300 × *g* at 4°C. The samples were stored at −80°C. The mice were sacrificed by cervical dislocation, and the middle colonic tissues and content (colonic feces) were collected. Middle colonic tissues were removed from a region containing feces and were frozen immediately in liquid nitrogen and stored at –80°C. Colonic feces and blood were prepared according to a published procedure [19]. Colons were suspended in methanol (500 μl) with 50 μM internal standard and vortexed vigorously five times for 60 s using a MicroSmash MS-100R (Tomy Digital Biology Co., Ltd., Tokyo, Japan) at 4,000 rpm at 4°C. The resulting colon sample served as the crude metabolome.

### Capillary electrophoresis–time-of-flight mass spectrometry (CE-TOFMS)

Metabolomic measurements were performed using an Agilent Capillary Electrophoresis System, and data were processed by Huma Metabolome Technologies Inc. (Tsuruoka, Japan) according to a published method [20]. All pretreated metablome samples were centrifugally filtered through a 5-kDa cutoff filter Ultrafree-MC (Millipore). The values of the peaks were normalized to those of the internal standards methionine sulfone (cationic) and D-camphor-10-sulfonic acid (anionic), respectively.

### Ratio of values in GF mice to those in Ex-GF mice (Ex-GF/GF ratio)

In this study, we calculated the ratio of values in GF mice to those in Ex-GF mice (Ex-GF/GF ratio) and classified the metabolites. We defined the threshold Ex-GF/GF ratio to determine whether metabolites were present at higher or lower concentrations in Ex-GF mice than in GF mice. The metabolites were measured in the metabolome of the cardiac plasma, which was influenced by all gateways: (i) the membrane transport system of colonocytes, (ii) metabolism during transcellular transport, and (iii) hepatic metabolism (Fig 1). The highest Ex-GF/GF ratio for metabolites present at higher concentrations in Ex-GF than in GF mice (where the difference was still significantly different) was 1.259 (S1 Table). The lowest Ex-GF/GF ratio for metabolites present at lower concentrations in Ex-GF than in GF mice was 0.866 (S1 Table). Therefore, in this study, the Ex-GF/GF ratio of metabolites present at higher concentrations in Ex-GF mice than in GF mice was defined as ≥1.25, and the Ex-GF/GF ratio of metabolites present at lower concentrations in Ex-GF mice than in GF mice was defined as < 0.8.

### Statistical analysis

IBM SPSS Statistics (Japan IBM, Tokyo) software was used to conduct statistical analyses. Metabolome data were analyzed using PCA and clustering analysis. Differences in relative quantities between GF and Ex-GF mice were evaluated for each metabolite using Welch’s *t* test. The levels of individual metabolites in GF and Ex-GF mice were compared using Fisher’s exact test.

## Results

### Analysis of the metabolomes of the colonic feces, colon, portal plasma, and cardiac plasma of GF and Ex-GF mice

**Fig 2:**
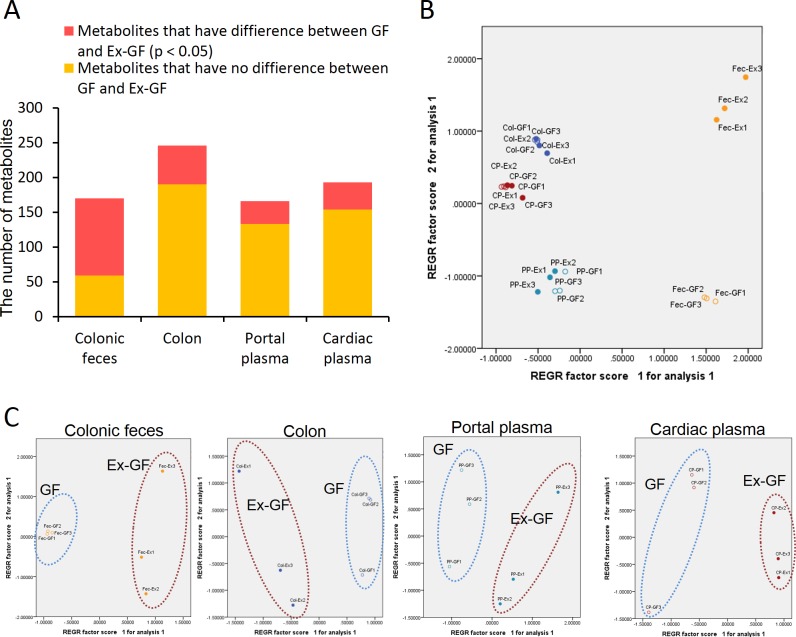
Differences in the metabolomes of GF and Ex-GF mice. (A) Number of metabolites that were significantly (*p* < 0.05) or not significantly different between GF and Ex-GF mice.(B) PCA of metabolome profiles. Fec-Ex, colonic feces of Ex-GF mice; Fec-GF, colonic feces of GF mice; Col-Ex, colon of Ex-GF mice; Col-GF, colon of GF mice; PP-Ex, portal plasma of Ex-GF mice; PP-GF, portal plasma of GF mice; CP-Ex, cardiac plasma of Ex-GF mice; CP-GF, cardiac plasma of GF mice.(C) PCA of metabolome profiles in each specimen.

CE-TOFMS identified 170, 246, 166, and 193 metabolites from the colonic feces, colonic tissue, portal plasma, and cardiac plasma, respectively. Significant differences (*p* < 0.05) were observed between GF and Ex-GF mice in each specimen; the number of metabolites with differences in the Ex-GF/GF ratio was as follows: colonic feces, 111 (65.7%); colonic tissue, 56 (22.8%); portal plasma, 33 (19.9%); and cardiac plasma, 39 (20.2%) (Fig 2A). Principal component analysis (PCA) showed patterns for metabolites in all samples (Fig 2B). The metabolomic profiles of colonic feces showed the greatest difference between the two groups of mice. However, the metabolome difference for colonic tissue, portal and cardiac plasma was not significant. Based on the PCA results of each specimen, it was possibles to assign all metabolomic profiles into two clusters corresponding to GF mice and Ex-GF mice (Fig 2C).

### Classification of metabolites according to the Ex-GF/GF ratio

In the present study, 146 colonic fecal metabolites detected in all mice were selected for analysis (122 metabolites that were detected in colonic feces and colonic tissue are shown in Table 1, and 24 metabolites that were detected in colonic feces but not in colonic tissue are shown in Table 2). The table shows that the levels of most of the selected metabolites were influenced by the intestinal microbiome in the colonic lumen because they showed significant differences between GF and Ex-GF mice. Transport of low-molecular-weight metabolites to the cardiac blood from the colonic lumen appeared to be influenced by 3 gateways: (i) the membrane transport system of colonocytes, (ii) metabolism during transcellular transport, and (iii) hepatic metabolism (Fig 1). Therefore, we estimated the effect of these gateways for each metabolite using the Ex-GF/GF ratio (Table 1). In total, 62 metabolites (No. 1 to No. 62) had Ex-GF/GF ratios that were similar between the colonic feces and colonic tissue, indicating that their levels are not influenced by the membrane transport system of colonocytes. Twenty-two metabolites (No. 1 to No. 22) had similar Ex-GF/GF ratios for colonic feces, colonic tissue, portal plasma, and cardiac plasma, and were thus probably transported from the colonic lumen to the systemic blood supply independently of factors (i), (ii), and (iii) defined above. The other 40 metabolites (No. 23 to No. 62), which had Ex-GF/GF ratios that were similar between the colonic feces and colonic tissue but not portal blood, were transported to colonic tissue, although their levels were controlled by (ii) metabolism during transcellular transport and/or (iii) hepatic metabolism. In contrast, 60 metabolites (No. 63 to No. 122) with different Ex-GF/GF ratios between the colonic feces and colonic tissue were also detected (Table 1). Because these metabolites are influenced by (i) the membrane transport system of colonocytes, their concentrations in the colonic tissue were independent of their concentrations in the colonic lumen. Twenty-four metabolites were not detected in the colonic tissue (Table 2). Therefore, these metabolites are not transported to the body from the colonic lumen. Eight of 11 peptides detected were in this group.

**Table 1:** The ExGF/GF ratio and the influence of 3 gateways on each metabolite detected in the colonic feces, colon, portal plasma, and cardiac plasma.

	Metbolites	Category		ExGF/GF ratio	Membrane transport system of colonocytes	ExGF/GF ratio	Metabolism during transcellular transport	ExGF/GF ratio	Hepatic metabolism	ExGF/GF ratio	
			KEGG ID	Colonic feces		Colon		Portal plasma		Cardiac plasma	
			(HMDB ID)								
1	Cytosine	Base/nucleotide	C00380		**→**	1.43	**→**		unclear	NA	
2	Cholic acid		C00695		**→**		**→**		**→**	1.71	
3	Pipecolic acid		C00408		**→**	2.45	**→**	1.61	**→**	1.66	
4	Stachydrine		C10172	1.14	**→**	0.94	**→**	0.84	**→**	0.90	
5	Phe*	Amino acid	C00079	0.76	**→**	0.64	**→**	0.76	**→**	0.83	
6	Val	Amino acid	C00183	0.66	**→**	0.66	**→**	0.61	**→**	0.64	
7	Lys	Amino acid	C00047	0.66	**→**	0.41	**→**	0.70	**→**	0.74	
8	Ile	Amino acid	C00407	0.64	**→**	0.56	**→**	0.57	**→**	0.60	
9	5-Hydroxylysine		C16741	0.60	**→**	0.50	**→**	0.59	**→**	0.72	
10	Leu	Amino acid	C00123	0.58	**→**	0.60	**→**	0.63	**→**	0.65	Ex-GF/GF
11	N8-Acetylspermidine*		C01029	0.51	**→**	0.37	**→**	0.67	**→/→**	0.82	3
12	Ser	Amino acid	C00065	0.50	**→**	0.36	**→**	0.56	**→**	0.59	2.5
13	*N*-Acetylhistidine		C02997	0.44	**→**	0.78	**→**	0.87	**→**	0.69	2
14	Gly	Amino acid	C00037	0.44	**→**	0.75	**→**	0.56	**→**	0.55	1.5
15	His	Amino acid	C00135	0.39	**→**	0.43	**→**	0.76	**→**	0.87	1
16	Thr	Amino acid	C00188	0.18	**→**	0.30	**→**	0.50	**→**	0.54	0.67
17	Arg	Amino acid	C00062	0.15	**→**	0.28	**→**	0.37	**→/→**	0.89	0.5
18	Pro	Amino acid	C00148	0.14	**→**	0.38	**→**	0.57	**→**	0.63	0.4
19	Hydroxyproline	Amino acid	C01015	0.05	**→**	0.22	**→**	0.47	**→**	0.54	0
20	Creatinine		C00791	0.01	**→**	0.34	**→**	0.66	**→**	0.77	
21	N-Acetyl-β-alanine	Alkylamino acid	C01073		**→**	0.40	**→**	0.73	**→/→**	1.52	
22	1-Methylnicotinamide	Alkylamino acid	C02918		**→**	0.51	**→**	0.50	**→**	0.45	
23	Glutaric acid		C00489		**→**		unclear	NA	**X**	ND	
24	Valeric acid	Fatty acid	C00803		**→**	39.53	**→/→**	1.22	**X**	ND	
25	2-Aminobutyric acid	Amino acid	C02261		**→**	1.89	**→/→**	0.73	**→**	0.78	
26	Sarcosine	Choline	C00213		**→**	2.43	unclear	NA	unclear	0.42	
27	1-Methyl-4-imidazoleacetic acid	Alkylamino acid	C05828	19.20	**→**	7.02	**→/→**	1.27	**→**	1.19	
28	Lactic acid	Energy	C00186	1.04	**→**	0.93	**→/→**	1.29	**→**	1.19	
29	Thiamine	Co-enzyme/its derivatives	C00378	0.98	**→**	1.18	**→/→**	1.73	**→**	1.75	
30	5-Oxoproline		C01879	0.88	**→**	0.75	**→/→**	1.43	**→**	2.11	
31	Trp	Amino acid	C00078	0.83	**→**	0.89	**→/→**	0.75	**→/→**	0.88	
32	Glu	Amino acid	C00025	0.78	**→**	0.89	**→/→**	1.94	**→**	1.29	
33	Carnitine		C00318	0.26	**→**	0.69	**→/→**	0.99	**→**	1.02	
34	Glucosamine		C00329	0.05	**→**	0.09	unclear	NA	unclear	NA	
35	Gluconic acid		C00257	0.01	**→**	0.20	**→/→**	1.52	**→/→**	1.10	
36	4-Guanidinobutyric acid		C01035		**→**	0.19	**→/→**	0.86	**→**	0.86	
37	Urea		C00086		**→**	0.71	**→/→**	0.96	**→**	0.99	
38	Ophthalmic acid	Peptide	(05765)		**→**	0.21	unclear	NA	unclear	0.47	
39	Asp	Amino acid	C00049	1.24	**→**	1.10	**→/→**	2.04	**→**	1.35	
40	2'-Deoxycytidine		C00881	0.18	**→**	0.56	**→/→**	1.23	**→**	1.16	
41	5-Methoxyindoleacetic acid	Alkylamino acid	C05660		**→**	0.67	**→/→**	1.31	**X**	ND	
42	S-Adenosylmethionine	Co-enzyme/its derivatives	C00019		**→**	0.74	unclear	NA	unclear	1.18	
43	Nicotinic acid	Co-enzyme/its derivatives	C00253	33.97	**→**		**X**	ND	unclear	ND	
44	5-Aminovaleric acid	Amino acid	C00431		**→**	32.55	**X**	ND	unclear	ND	
45	2,6-Diaminopimelic acid	Amino acid	C00666		**→**		**X**	ND	unclear	ND	
46	p-Hydroxyphenylacetic acid		C00642		**→**		**X**	ND	unclear	ND	
47	1H-Imidazole-4-propionic acid		-		**→**		**X**	ND	unclear	ND	
48	Piperidine		C01746		**→**		**X**	ND	unclear	ND	
49	Adenine		C00147		**→**	1.72	**X**	ND	unclear	ND	
50	Prostaglandin E2	Neurotransmitter	C00584		**→**	1.66	**X**	ND	unclear	ND	
51	N-Acetylglutamic acid	Alkylamino acid	C00624		**→**	1.62	**X**	ND	unclear	0.99	
52	4-Pyridoxic acid		C00847	4.46	**→**		**X**	ND	unclear	ND	
53	N-Acetylornithine	Alkylamino acid	C00437	2.17	**→**	1.62	**X**	ND	unclear	ND	
54	N-Acetylputrescine		C02714	1.51	**→**	1.28	**X**	ND	unclear	ND	
55	Methionine sulfoxide		C02989	1.01	**→**	1.18	**X**	ND	unclear	0.49	
56	Thr-Asp	Peptide	-	0.76	**→**	0.48	**X**	ND	unclear	ND	
57	Ser-Glu	Peptide	-	0.73	**→**	0.47	**X**	ND	unclear	ND	
58	SDMA		(03334)	0.57	**→**	0.59	**X**	ND	unclear	1.07	
59	N6,N6,N6-Trimethyllysine	Alkylamino acid	C03793	0.24	**→**	0.11	**X**	ND	unclear	ND	
60	Guanosine		C00387	0.12	**→**	0.69	**X**	ND	unclear	NA	
61	N-Acetylmethionine	Alkylamino acid	C02712		**→**	0.57	**X**	ND	unclear	ND	
62	Gly-Asp	Peptide	-		**→**	0.47	**X**	ND	unclear	ND	
63	β-Ala	Amino acid	C00099		**→/→**	1.11	**→**	1.18	**→**	1.24	
64	Pantothenic acid	Co-enzyme/its derivatives	C00864		**→/→**	0.85	**→**	1.07	**→/→**	1.45	
65	Taurine	Amino acid	C00245	16.86	**→/→**	1.19	**→**	1.08	**→**	1.09	
66	Citrulline	Amino acid	C00327	9.98	**→/→**	0.68	**→**	0.68	**→**	0.73	
67	Ornithine	Amino acid	C00077	7.38	**→/→**	0.94	**→**	1.18	**→/→**	0.69	
68	3-Methylhistidine	Alkylamino acid	C01152	2.52	**→/→**	1.04	**→**	0.90	**→**	0.94	
69	Pyridoxal		C00250	1.82	**→/→**	1.05	**→**	0.92	**→**	0.97	
70	N6-Acetyllysine	Alkylamino acid	C02727	1.39	**→/→**	0.46	**→**	0.51	**→**	0.68	
71	Met	Amino acid	C00073	1.02	**→/→**	0.41	**→**	0.52	**→**	0.57	
72	Isethionic acid		C05123	1.00	**→/→**	0.69	**→**	0.65	**→**	0.45	
73	Tyr	Amino acid	C00082	0.98	**→/→**	0.66	**→**	0.70	**→**	0.75	
74	Ala	Amino acid	C00041	0.90	**→/→**	0.79	**→**	0.66	**→**	0.57	
75	Gly-Leu	Peptide	C02155	0.86	**→/→**	0.25	**→**	0.66	**→**	0.47	
76	Choline	Choline	C00114	0.26	**→/→**	1.24	**→**	0.94	**→/→**	1.46	
77	Taurocholic acid		C05122	0.26	**→/→**	1.01	**→**	1.19	**→/→**	0.18	
78	Betaine	Choline	C00719	0.49	**→/→**	0.99	**→**	0.82	**→**	0.84	
79	Trimethylamine N-oxide		C01104		**→/→**	2.54	**→**	2.23	**→**	2.99	
80	O-Acetylcarnitine		C02571	0.14	**→/→**	0.88	**→**	1.20	**→**	1.19	
81	1-Methyladenosine		C02494		**→/→**	0.82	**→**	0.89	**→**	1.06	
82	Nicotinamide	Co-enzyme/its derivatives	C00153		**→/→**	0.89	**→**	0.98	**→/→**	0.62	
83	N-Acetylaspartic acid	Neurotransmitter	C01042		**→/→**	1.21	unclear	NA	**X**	ND	
84	N,N-Dimethylglycine	Choline	C01026		**→/→**	0.93	**→/→**	0.65	**→**	0.70	
85	Succinic acid	Energy	C00042		**→/→**	1.00	**→/→**	2.18	**→/→**	0.88	
86	3-Phenylpropionic acid		C05629		**→/→**	0.67	**→/→**	1.13	**X**	ND	
87	Cytidine		C00475		**→/→**	0.72	**→/→**	1.65	**→**	1.32	
88	Ribulose 5-phosphate	Energy	C00199		**→/→**	0.81	**→/→**	1.29	**→/→**	1.17	
89	Homoserine	Amino acid	C00263		**→/→**	0.91	unclear	NA	unclear	0.82	
90	Putrescine		C00134	17.15	**→/→**	1.06	unclear	NA	unclear	1.02	
91	γ-Butyrobetaine		C01181	8.97	**→/→**	1.08	**→/→**	0.53	**→**	0.57	
92	Glyceric acid		C00258	7.33	**→/→**	0.49	**→/→**	1.10	**X**	ND	
93	Hypoxanthine		C00262	5.01	**→/→**	0.94	**→/→**		**→**		
94	GABA	Neurotransmitter	C00334	3.66	**→/→**	1.11	**→/→**	1.26	**→**	4.20	
95	Urocanic acid		C00785	3.52	**→/→**	1.02	**→/→**	1.30	**→/→**	0.57	
96	Spermidine		C00315	2.85	**→/→**	0.96	unclear	NA	unclear	0.57	
97	Glycerol 3-phosphate		C00093	0.53	**→/→**	1.08	**→/→**	1.92	**→/→**	0.98	
98	Uric acid		C00366	0.44	**→/→**	1.00	**→/→**	1.54	**→/→**	0.80	
99	Gln	Amino acid	C00064	0.27	**→/→**	1.01	**→/→**	2.09	**→**	1.80	
100	Creatine		C00300	0.22	**→/→**	1.07	**→/→**	1.42	**→/→**	1.14	
101	Inosine		C00294	0.16	**→/→**	0.82	**→/→**		**→**		
102	Uridine		C00299		**→/→**	1.03	unclear	NA	unclear	1.55	
103	Cystine	Peptide	C00491		**→/→**	NA	**→/→**	0.47	**→**	0.59	
104	N-Methylproline	Alkylamino acid	-		**→/→**	NA	**X**	ND	unclear	ND	
105	Guanine		C00242		**→/→**	0.85	**X**	ND	unclear	ND	
106	Arg-Glu	Peptide	-		**→/→**	0.48	**X**	ND	unclear	ND	
107	β-Ala-Lys	Peptide	C05341		**→/→**	0.37	**X**	ND	unclear	ND	
108	Tyramine	Co-enzyme/its derivatives	C00483		**→/→**	1.19	**X**	ND	unclear	NA	
109	Uracil		C00106		**→/→**	1.04	**X**	ND	unclear	NA	
110	CMP		C00055		**→/→**	1.09	**X**	ND	unclear	1.07	
111	Propionic acid	Fatty acid	C00163		**→/→**	NA	**X**	ND	unclear	1.03	
112	Adenosine		C00212		**→/→**	0.53	**X**	ND	unclear	NA	
113	N-Acetylglucosamine	Alkylamino acid	C00140	7.41	**→/→**	1.01	**X**	ND	unclear	1.14	
114	N-Acetylneuraminic acid	Alkylamino acid	C00270	2.95	**→/→**	1.25	**X**	ND	unclear	0.57	
115	3-Aminoisobutyric acid	Amino acid	C05145	2.26	**→/→**	0.81	**X**	ND	unclear		
116	Glu-Glu	Peptide	C01425	0.83	**→/→**	NA	**X**	ND	unclear	ND	
117	His-Glu	Peptide	-	0.55	**→/→**	NA	**X**	ND	unclear	ND	
118	Betaine aldehyde_+H2O	Choline	C00576	0.41	**→/→**	1.74	**X**	ND	unclear	ND	
119	Spermine		C00750	0.28	**→/→**	0.95	**X**	ND	unclear	ND	
120	S-Lactoylglutathione	Peptide	C03451		**→/→**	3.11	**X**	ND	unclear	ND	
121	Thymidine		C00214		**→/→**	1.04	**X**	ND	unclear	NA	
122	Glucuronic acid		C00191		**→/→**	NA	**X**	ND	unclear	0.70	

**→**: metabolites are transported without selection by the gateway. **→/→**: metabolites are influenced by selection by the gateway. **X**: metabolites are blocked by the gateway. Metabolites detected in only Ex-GF or GF mice are indicated in red or green without the number, respectively.

*Although the Ex-GF/GF ratio was >0.8 in cardiac plasma, the ratio was judged as similar, because there was no significant difference between portal and cardiac plasma.

**Table 2:** Metabolites detected in colonic feces but not in colonic tissue.

Metbolites			ExGF/GF ratio				
	Category	KEGG ID	Colonic feces	Colon	Portal plasma	Cardiac plasma	
		
4-Methylbenzoic acid		-	20.00	ND	ND	ND	
Hydroxyindole		C03766	20.00	ND	ND	ND	Ex-GF/GF
3'-CMP; Cytidine 2'-monophosphate	Base/nucleotide	C03104	20.00	ND	ND	ND	3
Pyridoxamine		C13710	20.00	ND	ND	ND	2.5
1,3-Diaminopropane		-	20.00	ND	ND	ND	2
Cadaverine		C01672	20.00	ND	ND	ND	1.5
3-Methylbenzoic acid		C01454	20.00	ND	ND	ND	1
3-(4-Hydroxyphenyl)propionic acid		C01456	20.00	ND	ND	ND	0.67
Saccharopine		C00449	1.70	ND	ND	ND	0.5
Indole-3-acetamide		-	1.25	ND	ND	ND	0.4
Oxypurinol		C07599	1.21	ND	ND	ND	0
Allantoin		-	0.12	ND	ND	ND	
Galactosamine		C16675	0.09	ND	ND	ND	
3-Nitrotyrosine		-	0.00	ND	ND	ND	
O-Succinylhomoserine		C03776	0.00	ND	ND	ND	
γ-Glu-2-aminobutanoic acid			0.00	ND	ND	ND	
3-Hydroxy-3-methylglutaric acid		C03761	0.00	ND	ND	ND	
Glucaric acid		C00767	0.00	ND	ND	ND	
Quinic acid		C00296	0.00	ND	ND	ND	
2'-Deoxyguanosine	Base/nucleotide	C08507	0.41	ND	ND	ND	
2-Oxoglutaric acid		C00026	20.00	ND	1.03	ND	
4-Methyl-2-oxovaleric acid/ 3-Methyl-2-oxovaleric acid	Alkylamino acid	C00233	20.00	ND	0.70	ND	
Xanthine	Base/nucleotide	C00385	1.42	ND	ND	1.18	
Butyric acid	Fatty acid	C00246	20.00	ND	1.26	ND	

Metabolites detected in only Ex-GF or GF mice are indicated red or green without number, respectively.

## Discussion

This is the first study to clarify the effect of both the absorption system and intestinal microbiome on the bioavailability of colonic luminal low-molecular-weight metabolites. First, we explain why we believe this method was able to accurately detect the transport of metabolites from colonic lumen. If the colon does not contribute to the absorption of colonic luminal metabolites, as required by the notion that the colon absorbs only water, minerals, and some vitamins, the difference in the metabolite concentration in colonic tissue, portal plasma, and cardiac plasma between GF mice and Ex-GF mice would not be apparent. However, in the present study, we detected differences in the concentration of each metabolite between GF and Ex-GF mice in all specimens, and these Ex-GF/GF ratios were similar among the colonic feces, colonic tissue, portal plasma, and cardiac plasma. Thus, it is evident that this method is able to accurately detect low-molecular-weight metabolites transported to the body from the colonic lumen. In fact, the similarity of the Ex-GF/GF ratio of Arg, which is known to be absorbed by colonic epithelial cells [21], between colonic feces and colonic tissue supports this method.

The Ex-GF/GF ratio of 62 metabolites (No. 1 to No. 62) was similar between the colonic feces and colonic tissue, indicating that many metabolites are bioavailable and revising the general concept that the role of the colon is mainly to absorb water [22], electrolytes [22], and some vitamins [23]. In fact, some metabolites belonging to this group have been known as substances specifically absorbed from the colon, for example, amino acids [21, 24]. Furthermore, 56 metabolites (all of the metabolites except for stachydrine, lactic acid, thiamine, 5-oxoproline, Trp, and methionine sulfoxide) showed differences between GF and Ex-GF in colonic feces, indicating that many low-molecular-weight metabolites influenced by the intestinal microbiome are bioavailable.

From the classification of metabolites based on the Ex-GF/GF ratio of each specimen, we estimated the influence of these metabolites in health and disease. Twenty-two metabolites (No. 1 to No. 22) (Table 1), including 11 basic amino acids, that had similar Ex-GF/GF ratios for all specimens were transported to the systemic circulation from the colonic lumen. Therefore, these metabolites are probably directly involved in health and disease. Forty metabolites (No. 23 to No. 62) with similar Ex-GF/GF ratios between the colonic feces and colonic tissue but not portal blood are proposed to be key factors that influence colonic barrier function and the intestinal mucosal immune system. In contrast, 60 metabolites (No. 63 to No. 122) with different Ex-GF/GF ratios between the colonic feces and colonic tissue are stringently regulated by cellular homeostasis. For example, significant differences were observed in the Ex-GF/GF ratio of GABA (No. 94) between colonic feces and colonic tissue, and between portal plasma and cardiac plasma. Therefore, GABA must be regulated by the membrane transport system of colonocytes and by the hepatic metabolism because it functions as a neurotransmitter. Significant differences were also observed in the Ex-GF/GF ratios of Glu (No. 32), Asp (No. 39), and Tyr (No. 73), which function as neurotransmitters or precursors of neurotransmitters, in colonic feces and other specimens, although many basic amino acids are transported from the colonic lumen to the systemic blood with the same Ex-GF/GF ratio. Twenty-four metabolites that were not detected in the colonic tissue (Table 2) are not transported to the body from the colonic lumen. Eight of 11 peptides detected were in this group, indicating that nearly all peptides are not absorbed by colonocytes, although previous studies suggested that peptides are absorbed through peptide transporter 1 (PEPT1) [25, 26]. However, it was reported that PEPT1 is present in the distal part of the colon but not in the proximal colon [26]. Unfortunately, since the middle colon was analyzed in this study, the presence of PEPT1 is unclear. Surprisingly, butyrate, which is involved in the metabolism and normal development of colonic epithelial cells [27, 28], also belongs to this group, indicating that butyrate absorbed from the lumen is immediately metabolized in colonocytes because *in vivo* studies demonstrated that butyrate in the colonic lumen is absorbed and is metabolized to CO_2_, 3-hydroxybutyrate, and lactate [29, 30]. Colonic gene expression of butyrate transporter SLC5A8 is markedly lower in GF mice than in conventional mice [31], indicating that the stimulation of intestinal bacteria is an important factor in the expression of transporter. On the other hand, Jakobsdottir et al. [32] reported that the concentrations of acetate, propionate, and butyrate in the cecal content correlated significantly with those in portal serum after the oral administration of dietary fiber. Based on these reports, parts of the colon (distal, middle, and proximal) and effects of bacterial stimulation are also important factors to understand the colonic transport system of low-molecular-weight metabolites. Further study is required to clarify the bioavailability of peptides and SCFA in colonic tissue considering these points and using analytical instruments such as liquid chromatography for SCFA. Regarding polyamines, Ex-GF/GF ratios of fecal putrescine, spermidine, and spermine were 17.15, 2.85, and 0.28, respectively (Table 1), demonstrating that the colonic luminal putrescine and spermidine are produced by the colonic microbiome. In contrast, colonic luminal spermine is probably derived from live or exfoliated epithelial cells and is absorbed by the colonic microbiome. On the other hand, since polyamines have to be strictly controlled because of physiological function and toxicity in the body, Ex-GF/GF ratios of all polyamines in colon tissue were around 1 (putrescine: 1.06, spermidine: 0.96, and spermine: 0.95).

To the best of our knowledge, little is known about the colonic transporter that absorbs metabolites. Although it is known that lipophiles are delivered by passive diffusion across the enterocyte membrane [33],water-soluble substances detected using CE-TOFMS are probably absorbed through transporters using energy produced by Na^+^/K^+^ATPase. For example, amino acids are absorbed through the Na^+^ pump transporter [34]. Here, we show that 14 basic amino acids were absorbed into colonocytes (colonic tissue) with an Ex-GF/GF ratio similar to that of colonic feces, indicating that the transporters mediate the uptake of amino acids by colonocytes. The present study indicates that known/unknown transporters are used to absorb water-soluble metabolites.

**Fig 3:**
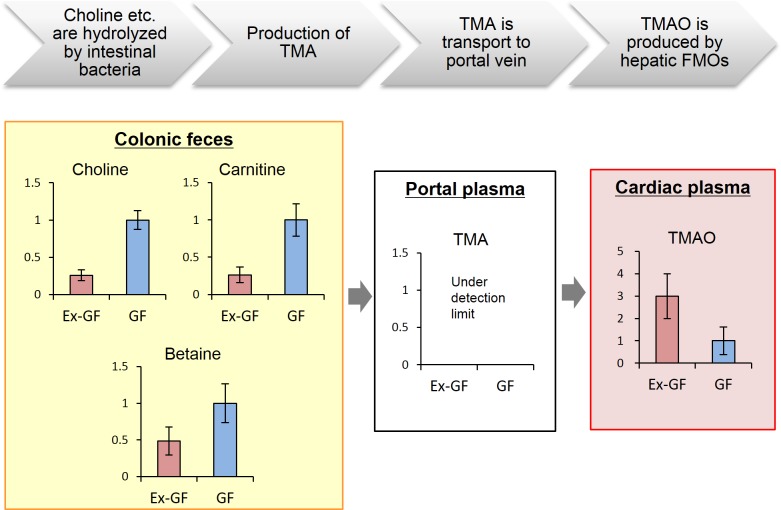
Metabolomic snapshots of the known phenomenon of intestinal microbiome-dependent production of pro-atherogenic trimethylamine *N*-oxide by degradation of carnitine/choline in the intestinal tract. FMO: flavin-containing monooxygenase.

We confirmed the validity of this study using snapshots of the metabolome corresponding to the known relationship among the intestinal microbiome, intestinal bacterial metabolites, and health/disease using bacterial metabolism of trimethylamine *N*-oxide (TMAO) from choline, which is associated with cardiovascular disease risk [35]. Here, we showed that the colonic fecal concentrations of choline (No. 76) and carnitine (No. 33) in Ex-GF mice were lower than those in GF mice because they are metabolized by the intestinal microbiota (Fig 3). Furthermore, the TMAO (No. 79) concentrations in the cardiac plasma were higher in Ex-GF mice because TMAO was produced through liver metabolism of trimethylamine (TMA) produced by the intestinal microbiota in Ex-GF mice (the TMA concentration was below the limit of detection). Therefore, we consider that the metabolome data obtained from the present study are useful for understanding the mechanism of diseases caused by intestinal microbiome.

In the present study, we succeeded in the estimation of low-molecular-weight metabolites transported to the rest of the body from the colonic lumen by focusing on differences in the Ex-GF/GF ratio between GF and Ex-GF mice and have thus revised the general concept of the role of the colon. We also found that many low-molecular-weight metabolites influenced by the intestinal microbiome appeared to be absorbed from the colonic lumen. In the field of gastrointestinal physiology, it is very important to note that many low-molecular-weight chemicals influenced by intestinal microbiome in the large intestine appeared to be absorbed from the colonic lumen. Although this study does not provide direct evidence that low-molecular-weight metabolites influenced by intestinal microbiome are absorbed into the body from the large intestine, it contributes to the field of nutrition by providing a list of low-molecular-weight metabolites that are transported to the body from the colonic lumen. In other fields such as medicine, immunology, physiology, pharmacology, bacteriology, and nutrition, these findings may also contribute new insight into the function of the colon and its interactions with the intestinal microbiome and provide candidate chemicals whose roles in the body can be elucidated in future studies. In addition, further studies are required to investigate the colonic absorption of low-molecular-metabolites in mice of various strains and ages and in female mice.

## Supporting Information

S1 Table All metabolites and their Ex-GF/GF ratio detected from cardiac plasma. (DOCX)
