# Supplementary material for: Colonic Absorption of Low-Molecular-Weight Metabolites Influenced by the Intestinal Microbiome: A Pilot Study
Source: PLoS One. 2017 Jan 25;12(1):e0169207. doi: 10.1371/journal.pone.0169207 (PMC5266324; doi:10.1371/journal.pone.0169207)
Supplement: S1 Table — (DOCX) [file pone.0169207.s001.docx]

S1 Table. All metabolites and their Ex-GF/GF ratio detected from cardiac plasma.

Supplemental Table 1.　Continued
